# Sample Size Requirements for Calibrated Approximate Credible Intervals for Proportions in Clinical Trials

**DOI:** 10.3390/ijerph18020595

**Published:** 2021-01-12

**Authors:** Fulvio De Santis, Stefania Gubbiotti

**Affiliations:** Dipartimento di Scienze Statistiche, Sapienza University of Rome, Piazzale Aldo Moro n. 5, 00185 Rome, Italy; fulvio.desantis@uniroma1.it

**Keywords:** Bayesian inference, highest posterior density intervals, normal approximation, predictive analysis, sample size determination

## Abstract

In Bayesian analysis of clinical trials data, credible intervals are widely used for inference on unknown parameters of interest, such as treatment effects or differences in treatments effects. Highest Posterior Density (HPD) sets are often used because they guarantee the shortest length. In most of standard problems, closed-form expressions for exact HPD intervals do not exist, but they are available for intervals based on the normal approximation of the posterior distribution. For small sample sizes, approximate intervals may be not calibrated in terms of posterior probability, but for increasing sample sizes their posterior probability tends to the correct credible level and they become closer and closer to exact sets. The article proposes a predictive analysis to select appropriate sample sizes needed to have approximate intervals calibrated at a pre-specified level. Examples are given for interval estimation of proportions and log-odds.

## 1. Introduction

The use of Bayesian methods for design, analysis and monitoring of clinical trials is becoming more and more popular. For instance, in some recent contributions [1,2] the Authors note that “compared with its frequentist counterpart, the Bayesian framework has several unique advantages, and its incorporation into clinical trial design is occurring more frequently.” Acknowledgements have been arriving also from official institutions. In 2010 FDA, recognizing the merits of Bayesian inference, authorized and encouraged its use in medical device clinical trials. Similarly Bittle and He observe that “ […] in a major shift, the American College of Cardiology and American Heart Association have recently proposed using Bayesian analysis to create clinical trials guidelines” [3].

There are at least two main motivations for using Bayesian methods. The first is that, unlike frequentist analysis, the Bayesian approach allows the integration of information from a current experiment with pre-trial knowledge. The second advantage is that Bayesian inferential methods are derived from probability distributions that are directly defined on the quantity of interest in the trial (i.e., the parameter). This makes communication between statisticians and experts in the field much more effective than it is when frequentist methods are employed.

With no significant loss of generality, suppose we are concerned with inference on the unknown effect of a new treatment, that we assume to be our parameter of interest. Bayesian methodology is based on elaborations of the posterior distribution of the parameter, which merges pre-experimental knowledge (i.e., the prior distribution) and trial information (i.e., the likelihood function) on this parameter via Bayes theorem. Inferential tools—such as point estimates, set estimates or test statistics—are simply special functionals of the posterior distributions. Nowadays analytic and computational methods for handling complex Bayesian problems are available, even in high dimensional settings. Nevertheless, the availability of closed-form expressions makes the use of Bayesian analysis more accessible also to non-statisticians. For this reason a relevant part of the available Bayesian literature in clinical trials resorts heavily to normal approximations [4].

Interval estimation is one of the most common techniques used to summarize information on an unknown parameter. Bayesian inference usually relies on exact Highest Posterior Density intervals (HPD). The (1−γ)-HPD interval is the subset of the parameter space of probability (1−γ) whose points have density higher than the density of any value of the parameter outside the interval. When the posterior distribution is symmetric, HPDs are also equal-tails (ET) intervals, i.e., they are limited respectively by the γ/2 and the 1−γ/2 quantiles of the posterior density of the parameter. HPDs are, typically, not easy to compute, but of minimal length among intervals of given credibility. For a predictive comparison between HPDs and ETs see [5]. Explicit closed-form expressions for the bounds of common exact credible intervals are in most of the cases, not available even in very common models. However, their computation can be simplified by approximating the exact posterior distribution with a normal density and finding the equal-tails intervals, i.e., the γ/2 and the 1−γ/2 quantiles of the approximated (symmetric) normal density.

**Figure 1:**
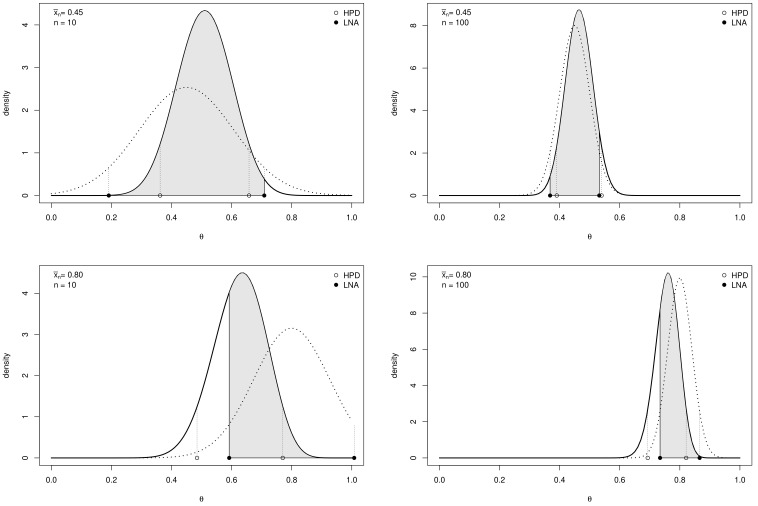
Posterior density, given a prior density of hyperparameters (α,β)=(10.8,9.2), and likelihood approximation, given ${\bar{x}}_{n}=0.45$ (*top row*) and ${\bar{x}}_{n}=0.8$ (*bottom row*) for n=10 (*left column*) and n=100 (*right column*). Exact credible intervals (HPD: Highest Posterior Density) are denoted by empty circles, likelihood approximated credible intervals (LNA: Likelihood Normal Approximation) are denoted by black circles. The probability that θ belongs to the approximate interval under the exact posterior distribution is highlighted in grey.

In many standard models the posterior density has a unique mode internal to its support. The degree of skewness of the posterior distribution with respect to its mode depends on the shapes of the likelihood function and of the prior distribution [6]. As shown in Figure 1 asymmetry affects the quality of approximate credible intervals that in general may differ substantially from exact HPDs. This means that, in general, for approximate intervals: (a) their actual posterior probability is not equal to the nominal credibility of the exact interval; (b) they are not the shortest intervals among those of given posterior probability.

Under standard and fairly general conditions [7], the degree of asymmetry of the likelihood function is strictly related to the sample size: as the number of experimental units increases, the shape of the likelihood becomes closer and closer to a Gaussian function whose mode is the maximum likelihood estimate and whose precision is measured by the square root of the observed Fisher Information [8]. Likelihood normalization carries along the same tendency of the posterior distribution and, for sufficiently large sample sizes, the posterior density can be approximated by a normal density with data-dependent parameters. This is the so-called Bayesian Central Limit Theorem. As a consequence, as the sample size increases, exact and approximate intervals become closer and closer and the accuracy of approximate intervals improves.

Example 1 (*Single  arm  phase  II  trial*). Let us consider an example for binary data, where θ is the probability of response to a treatment. The setup, the choice of the prior hyperparameters and a sensitivity analysis will be fully described in Section 4. A Beta prior of mean 0.54 is considered. Figure 1 shows the Beta posterior distributions of θ (solid line) and their normal approximations based on the likelihood (dotted line) for four different data sets. It also reports bounds of approximate intervals (black circle) and of exact HPD intervals (empty circles). Gray areas highlight the probability of the approximate intervals w.r.t. the exact posterior probability distributions. More specifically, when comparing right panels (n=100) and left panels (n=10) better approximations of the posteriors are observed, due to the larger sample size. Furthermore, the comparison between the two rows of panels (sample mean ${\bar{x}}_{n}=0.45$ and ${\bar{x}}_{n}=0.80$, respectively) shows that the distance between the posterior mode and the likelihood mode (i.e., the maximum likelihood estimate) affects the quality of the approximation: in this example, the larger the difference, the greater the discrepancy between exact and approximate intervals.

The problem we discuss in this paper is the selection of the minimal number of observations to obtain approximate sets that are sufficiently accurate. This sample size determination (SSD) problem is addressed from a pre-posterior perspective, i.e., by taking into account the randomness of the posterior density and of credible intervals.

In the existing literature besides a very general introduction to credible intervals  [6,7,9,10] one can find reviews on Bayesian SSD in [11,12,13], articles specifically dedicated to Bayesian SSD using credible intervals in [14,15,16,17] and some contributions focused on binomial proportions, such as [18,19,20]. Recently, methods that take into account the variability of prior opinion have been developed: for instance, some contributions [15,21,22] deal with robustness with respect to the prior distribution, whereas a more recent proposal is about a consensus-based SSD criterion in the presence of a community of priors [23]. The idea of controlling the conflict between alternative procedures is also used for point estimation  [24,25].

In the framework of Bayesian SSD based on credible intervals, our innovative purpose is to look for a sample size sufficiently large so that the approximate likelihood interval provides an accurate approximation to the HPD interval determined from the exact posterior distribution of the parameter of interest. It is worth recalling that whereas the HPD interval is obtained from the prior-to-posterior analysis, the likelihood normal approximation is independent on the prior distribution. In this sense our proposed criterion yields the smallest sample size such that the role of the prior in the posterior distribution is made negligible by the information provided by the data. This provides an additional motivation for our proposal, i.e., to find the study dimension that guarantees a substantial equivalence between closed-form formulas based on the normal approximation and exact Bayesian intervals, or, conversely, to evaluate the expected discrepancy between approximate intervals and exact Bayesian intervals.

The paper is organized as follows. In Section 2, after introducing notation, we propose a measure of discrepancy between exact and approximate intervals to be analyzed from a preposterior perspective: we select the minimal sample size so that the expected discrepancy is sufficiently small. Section 3 specifically refers to the Beta-Binomial model when the paramer of interest is the proportion (Section 3.1) and the logodds (Section 3.2) respectively. Section 4 illustrates some numerical examples related to the setup of the phase II clinical trial of Example 1 and makes comparison with other SSD methods. Finally, Section 5 contains some concluding remarks.

## 2. Methodology

Assume that $X_{1},X_{2},\ldots ,X_{n}$ is a sample from $f_{n}\left(\cdot |\theta \right)$ (either a density or a probability mass function), where θ∈Θ is an unknown scalar parameter and Θ is the parameter space. The quantity of interest may be either θ or a relevant function ψ=g(θ). Following the Bayesian inferential approach, we assume that prior information on θ is available (from experts or from historical data) and converted in a prior probability density function, denoted as π(·). Given an observed sample $x_{n}=\left(x_{1},x_{2},\ldots ,x_{n}\right)$, let

$$\pi \left(\theta |x_{n}\right)=\frac{f_{n}\left(x_{n}|\theta \right)\pi \left(\theta \right)}{m(x_{n})}$$

be the posterior distribution of θ, where $m\left(x_{n}\right)=\int_{\Theta }f_{n}\left(x_{n}|\theta \right)\pi \left(\theta \right)d\theta$ denotes the marginal distribution of the the data, computed at the observed $x_{n}$. In the following we assume that $\pi (\theta |x_{n})$ has a unique mode.

### 2.1. Exact and Approximate Intervals

Let $C\left(x_{n}\right)=\left[\ell \left(x_{n}\right),u\left(x_{n}\right)\right]$ be an exact credible interval of level 1−γ, that is a subset of the parameter space such that

(1) $$P[\theta \in C\left(x_{n}\right)|x_{n}]=1-\gamma .$$

In the following, we will focus on HPD intervals. *C* is HPD if

$$\pi \left(\theta |x_{n}\right)\geq \pi \left(\theta^{\prime }|x_{n}\right),\;\forall \theta \in C\left(x_{n}\right)\;\mathrm{and}\;\forall \theta^{\prime }\notin C\left(x_{n}\right),$$

or, equivalently, if

$$C\left(x_{n}\right)=\left\{\theta \in \Theta :\pi \left(\theta |x_{n}\right)\geq k_{\gamma }\right\},$$

where $k_{\gamma }$ is such that (1) holds. The values of *ℓ* and *u* are the roots of the two equations

$$\pi \left(\ell |x_{n}\right)=\pi \left(u|x_{n}\right)\;\mathrm{and}\;\int_{\ell }^{u}\pi \left(\theta |x_{n}\right)d\theta =1-\gamma ,$$

and they typically do not have a closed-form expression.

In general, $\pi (\theta |x_{n})$ is not symmetric with respect to its unique mode. Its level of skewness depends on the constitutive elements of Bayesian analysis—the likelihood (i.e., model and observed data) and the prior distribution— and it determines the level of discrepancy between approximate and exact credible intervals. However, as the sample size increases, the shape of both the likelihood function and the posterior density tend to become more and more Gaussian. This happens under standard regularity conditions: (a) the support of the $X_{i}$’s does not depend on θ; (b) the derivatives with respect to θ of likelihood and posterior density at least up to the second order exist; (c) the maximum likelihood estimate of θ, $\hat{\theta }$, is in the interior of the parameter space [6,7,8]. More specifically, for sufficiently large *n* we have that

(2) $$\theta |x_{n}\approx N\left[\hat{\theta },I_{n}{\left(\hat{\theta }\right)}^{-1}\right],$$

where $I_{n}\left(\theta \right)=-\frac{d^{2}}{d\theta^{2}}\ln L\left(\theta ;x_{n}\right)$ is the expected Fisher Information and $L(\theta ;x_{n})$ is the likelihood function. Note that this approximation of the posterior distribution does not take into account the prior. From Equation (2) the (1−γ)-*likelihood approximate* interval for θ is defined as $\tilde{C}\left(x_{n}\right)=\left[\tilde{\ell }\left(x_{n}\right),\tilde{u}\left(x_{n}\right)\right]$ where

(3) $$\tilde{\ell }=\hat{\theta }-z_{1-\frac{\gamma }{2}}I_{n}{\left(\hat{\theta }\right)}^{-1/2}\;\mathrm{and}\;\tilde{u}=\hat{\theta }+z_{1-\frac{\gamma }{2}}I_{n}{\left(\hat{\theta }\right)}^{-1/2},$$

with $z_{\epsilon }$ denoting the ϵ-quantile of the standard normal distribution. As a consequence, as *n* increases, any measure of discrepancy between a chosen feature of exact and approximate intervals tends to become more and more negligible.

When the quantity of interest is ψ=g(θ), under the same regularity conditions stated above and assuming that the first derivative of *g* exists and is not equal to 0, the delta method provides the following normal approximation [26]

(4) $$\psi |x_{n}\approx N\left[g\left(\hat{\theta }\right),g^{\prime }{\left(\hat{\theta }\right)}^{2}\;I_{n}{\left(\hat{\theta }\right)}^{-1}\right],$$

and the bounds of the (1−γ) *likelihood approximate* credible interval for ψ are respectively

(5) $$\tilde{\ell }=g\left(\hat{\theta }\right)-z_{1-\frac{\gamma }{2}}\cdot |g^{\prime }\left(\hat{\theta }\right)|\cdot I_{n}{\left(\hat{\theta }\right)}^{-1/2}\;\;\;\;\mathrm{and}\;\;\;\;\tilde{u}=g\left(\hat{\theta }\right)+z_{1-\frac{\gamma }{2}}\cdot \left|g^{\prime }\left(\hat{\theta }\right)\right|\cdot I_{n}{\left(\hat{\theta }\right)}^{-1/2}.$$

### 2.2. A Measure of Discrepancy and Predictive Analysis

The set $\tilde{C}=\left[\tilde{\ell },\tilde{u}\right]$ is calibrated if its exact posterior probability is equal to 1−γ:

(6) $$\begin{array}{c} P\left(\theta \in \tilde{C}|x_{n}\right)=F\left(\tilde{u}|x_{n}\right)-F\left(\tilde{\ell }|x_{n}\right)=\left(1-\gamma \right), \end{array}$$

where $F(\cdot |x_{n})$ is the exact posterior cumulative distribution function of the parameter of interest. The departure from this situation can be measured by

(7) $$\begin{array}{c} \left|P\left(\theta \in \tilde{C}|x_{n}\right)-\left(1-\gamma \right)\right| \end{array}$$

which quantifies the discrepancy between the actual posterior *probability* of $\tilde{C}$ (the gray area of each panel of Figure 1 in Example 1) and its nominal value 1−γ. Notice that, under the typical assumption $0<\gamma \ll \frac{1}{2}$, this discrepancy takes values in (0,1−γ). More specifically, it is equal to 0 when $\tilde{C}$ is perfectly calibrated and it is equal to 1−γ when $P(\theta \in \tilde{C}|x_{n})=0$. Hence, a relative measure based on (7) is

(8) $$\begin{array}{c} P\left(x_{n}\right)=\frac{\left|P\left(\theta \in \tilde{C}|x_{n}\right)-\left(1-\gamma \right)\right|}{1-\gamma } \end{array}$$

Before observing the data, $P(X_{n})$ is a random object. Therefore the progressive calibration of $\tilde{C}\left(X_{n}\right)$ can be studied by looking at its expected value

$$e_{n}^{P}=E_{d}\left[P\left(X_{n}\right)\right],$$

that is computed with respect to the sampling distribution of the data $f_{n}\left(\cdot |\theta_{d}\right)$ for a design value $\theta_{d}$. In the following we assume that all the required regularity conditions hold such that the numerical sequence $\left\{e_{n}^{P},n\in N\right\}$ converges to zero.

In order to obtain a calibrated approximate interval, we must select the smallest sample size such that $e_{n}^{P}$ is sufficiently small. More formally, for a suitable threshold $\epsilon_{P}>0$,

(9) $$n_{P}^{☆}=\min\left\{n\in N:e_{n}^{P}<\epsilon_{P}\right\}.$$

In some cases the values of $e_{n}^{P}$ can be obtained with exact calculations. More often they are obtained via Monte Carlo (MC) simulation. In the latter case, for each sample size *n* and design value $\theta_{d}$, we proceed according to the following steps:

(i) draw *N* samples ${x_{n}}^{\left(1\right)},\ldots ,{x_{n}}^{\left(N\right)}$ from $f_{n}\left(\cdot ;\theta_{d}\right)$;
(ii) compute $\tilde{\ell }\left({x_{n}}^{\left(j\right)}\right)$ and $\tilde{u}\left({x_{n}}^{\left(j\right)}\right)$, for j=1,…,N;
(iii) compute $P({x_{n}}^{\left(j\right)})$, for j=1,…,N;
(iv) set $e_{n}^{P}≃\frac{\sum_{j=1}^{N}P\left({x_{n}}^{\left(j\right)}\right)}{N}$;
  with a large number of draws, e.g., N=10000.

In the following example, in order to assess the discrepancy between $\tilde{C}$ and *C* we also consider the absolute distance between their *bounds*

$$B\left(x_{n}\right)=|\tilde{\ell }\left(x_{n}\right)-\ell \left(x_{n}\right)\left|+\right|\tilde{u}\left(x_{n}\right)-u\left(x_{n}\right)|$$

and we compare $n_{P}^{☆}$ with

(10) $$n_{B}^{☆}=\min\left\{n\in N:e_{n}^{B}<\epsilon_{B}\right\},$$

where

$$e_{n}^{B}=E_{d}\left[B\left(X_{n}\right)\right],$$

and $\epsilon_{B}>0$ is a chosen threshold. Note that, unlike $P(x_{n})$ (and $e_{n}^{P}$), the discrepancy $B(x_{n})$ (and $e_{n}^{B}$) depends on the unit of measurement of the data and its range is case-specific. Therefore the choice of $\epsilon_{B}$ is a critical issue, unless the parameter space is bounded (as in Example 1 where the parameter space is (0,1)). Similar measures of discrepancy based on the bounds of credible intervals have been recently proposed [23].

## 3. Examples: The Beta-Binomial Model

In order to illustrate the ideas sketched above we now consider an example within the Beta-Binomial model. Let $X_{i}|\theta ∼\mathrm{Ber}\left(\theta \right)$, i=1,…,n (i.i.d.), θ∈(0,1) and θ∼Be(α,β), α,β>0. Then, from standard results [6], $\theta |x_{n}∼\mathrm{Be}\left(\bar{\alpha },\bar{\beta }\right)$, where $\bar{\alpha }=\alpha +s_{n}$, $\bar{\beta }=\beta +n-s_{n}$ and $s_{n}=\sum_{i=1}^{n}x_{i}$. In the following we first analyze credible intervals for θ and then for the log-odds $\psi =g\left(\theta \right)=\ln\frac{\theta }{1-\theta }$.

### 3.1. Credible Intervals for a Proportion

In this model exact HPD credible intervals for θ do not have closed-form expressions. However, HPD bounds are easily obtained using the hdi() function of the HDInterval package of R, [27], which simply requires the R function qbeta() in input. Conversely, closed-form expressions for approximate intervals are easily obtained as follows. Recalling that $\hat{\theta }={\bar{x}}_{n}$ and $I_{n}\left(\theta \right)=\frac{n}{\theta (1-\theta )}$, from Equation (3) the bounds of the *likelihood approximate interval* are

$$\tilde{\ell }={\bar{x}}_{n}-z_{1-\frac{\gamma }{2}}\sqrt{\frac{{\bar{x}}_{n}\left(1-{\bar{x}}_{n}\right)}{n}}\;\mathrm{and}\;\tilde{u}={\bar{x}}_{n}+z_{1-\frac{\gamma }{2}}\sqrt{\frac{{\bar{x}}_{n}\left(1-{\bar{x}}_{n}\right)}{n}}.$$

### 3.2. Credible Intervals for the Log-Odds

As before, exact credible intervals for ψ do not have a closed-form expression. HPD bounds can be otained via MC simulation as follows:

(i) draw $\theta^{\left(1\right)},\ldots ,\theta^{\left(M\right)}$ from the posterior Beta density, where *M* is a large number;
(ii) compute $\psi^{\left(j\right)}=g\left(\theta^{\left(j\right)}\right)$, for j=1,…,M;
(iii) use the R function HDInterval::hdi with the MC draws $\psi^{\left(1\right)},\ldots ,\psi^{\left(M\right)}$ in input.

Closed-form expression of approximate credible intervals for ψ are obtained from Equation (5) noting that

$$g\left(\hat{\theta }\right)=\ln\frac{{\bar{x}}_{n}}{1-{\bar{x}}_{n}}\;\;\mathrm{and}\;\;g^{\prime }\left(\hat{\theta }\right)=\frac{1}{{\bar{x}}_{n}\left(1-{\bar{x}}_{n}\right)}.$$

Specifically, we have

$$\tilde{\ell }=\ln\frac{{\bar{x}}_{n}}{1-{\bar{x}}_{n}}-z_{1-\frac{\gamma }{2}}\cdot \sqrt{\frac{1}{n{\bar{x}}_{n}\left(1-{\bar{x}}_{n}\right)}}\;\mathrm{and}\;\tilde{u}=\ln\frac{{\bar{x}}_{n}}{1-{\bar{x}}_{n}}+z_{1-\frac{\gamma }{2}}\cdot \sqrt{\frac{1}{n{\bar{x}}_{n}\left(1-{\bar{x}}_{n}\right)}}.$$

Note that in the Beta-Binomial model the values of $e_{n}^{P}$ can be obtained using either exact calculations or MC simulations as described in Section 2.2.

## 4. Application to Clinical Trials

Let us assume that in an early phase trial we are interested in estimating the rate of response, θ, to an experimental treatment using a credible interval. As in Example 1 we consider the setup of a single-arm phase II trial. Specifically, the goal of the study is to test the combination of lenalidomideandrituximab in patients with recurrent indolent non-follicular lymphoma [28,29,30]. The endpoint is the overall response rate $\hat{\theta }$, that is the proportion of eligible patients who achieved complete, unconfirmed or partial response.

In the trial conducted between 2009 and 2011, 21 responses were observed out of 39 eligible patients. These hystorical data are used to elicit a Beta prior density for θ. More specifically, we set the prior mean equal to α/(α+β)=0.54 and we consider several values for the prior sample size (i.e., the amount of information contained in the prior) that for the Beta model is α+β [31]. For illustrative purposes in the following example we set α+β equal to 5, 10 and 20. Moreover, for comparison, we also consider a uniform density as non-informative prior (e.g., α=β=1). The design value $\theta_{d}$ is set equal to 0.45, that is the lowest acceptable value for the overall response rate [28]. In order to evaluate the impact of the design parameter we also consider $\theta_{d}=0.8$ that represents a much more optimistic design scenario.

**Figure 2:**
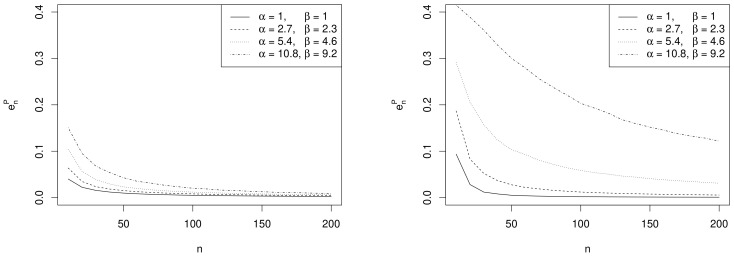
Plots of $e_{n}^{P}$ as a function of *n* for several values of the prior hyperparameters (α,β), with $\theta_{d}=0.45$ (**left column**) and $\theta_{d}=0.8$ (**right column**).

Figure 2 shows the behaviour of $e_{n}^{P}$ for increasing values of the sample size *n* under different prior assumptions. Table 1 reports the optimal sample sizes $n_{P}^{☆}$ and $n_{B}^{☆}$ obtained using criteria (9) and (10) for several choices of the prior hyperameters, when $\theta_{d}=0.45$ and $\theta_{d}=0.8$, given $\epsilon_{P}=\epsilon_{B}=0.01$ (i.e., 1% of the width of the parameter space). Table 1 also contains the optimal sample sizes obtained using the Average Length Criterion ALC [13], given a threshold for the interval width as small as 0.1, for both exact ($n_{L}^{☆}$) and approximate intervals ($n_{\tilde{L}}^{☆}$).

The most relevant comments are the following.

1. *Effect of sample size.* As expected, the values of $e_{n}^{P}$ decrease as *n* increases and depend on the specific choices of α, β and $\theta_{d}$ as commented in the following remarks.
2. *Effect of prior sample size.* For each value of *n*, the larger α+β, the greater the values of $e_{n}^{P}$. In fact, as the prior becomes more and more concentrated around the prior mean 0.54, the weight of the prior in the posterior distribution increases with respect to the role of the likelihood. This makes the discrepancy between Bayesian exact intervals and their likelihood approximation more striking. Moreover, when the uniform non-informative prior is considered, the smallest values of $e_{n}^{P}$ are observed (see solid line in Figure 2). As a consequence, larger values of the prior sample size imply greater values of $n_{P}^{☆}$, as shown in Table 1.
3. *Effect of the difference between design value and prior mean.* When the distance between $\theta_{d}$ and the prior mean α/(α+β) is relatively large and, at the same time, the prior sample size α+β dominates *n*, the posterior mode and the maximum likelihood estimate are well separated. In other words, Equation (4) does not provide a good approximation of the posterior density of θ. This explains the larger values of $e_{n}^{P}$, in the right panel of Figure 2, where $|\theta_{d}-E\left(\theta \right)|=0.35$, with respect to those observed in the left panel, where $|\theta_{d}-E\left(\theta \right)|=0.09$. As before, the effect of the difference between design value and prior mean on $e_{n}^{P}$ also reflects on the values of the optimal sample sizes reported in Table 1. For instance, under the most informative prior, if $|\theta_{d}-E\left(\theta \right)|=0.09$, then $n_{P}^{☆}=182$; conversely, when $|\theta_{d}-E\left(\theta \right)|=0.35$, a huge number of experimental units (e.g., $n_{P}^{☆}=2911$) is required to have a sufficiently small expected discrepancy.
4. *Comparison with $n_{B}^{☆}$.* As expected, the trend of $n_{B}^{☆}$ w.r.t. to (α,β) and $\theta_{d}$ is consistent with that of $n_{P}^{☆}$.
5. *Comparison with ALC*. For each $\theta_{d}$, $n_{L}^{☆}$ becomes slightly smaller when the prior sample size gets larger and the corresponding posterior is more concentrated (see Table 1). Conversely, since approximate intervals do not depend on the prior, $n_{\tilde{L}}^{☆}$ is not affected by the choice of prior hyperparameters. Furthermore, when the design value is closer to the boundary of the parameter space, the posterior distribution and, consequently, its approximation, become more concentrated, yielding shorter intervals. Hence the values of $n_{L}^{☆}$ and of $n_{\tilde{L}}^{☆}$ are uniformly smaller for $\theta_{d}=0.80$ than for $\theta_{d}=0.45$.
  It is interesting to note the opposite impact of the prior sample size α+β on $n_{P}^{☆}$ and $n_{B}^{☆}$ on the one hand, and on $n_{L}^{☆}$ on the other hand. In fact, larger values of α+β determine shorter intervals and smaller values of $n_{L}^{☆}$. On the contrary, when $\theta_{d}\neq E\left(\theta \right)$, a more concentrated prior implies a more remarkable discrepancy between the posterior and its likelihood approximation and, consequently, yields greater values of $n_{P}^{☆}$ and $n_{B}^{☆}$.

**Figure 3:**
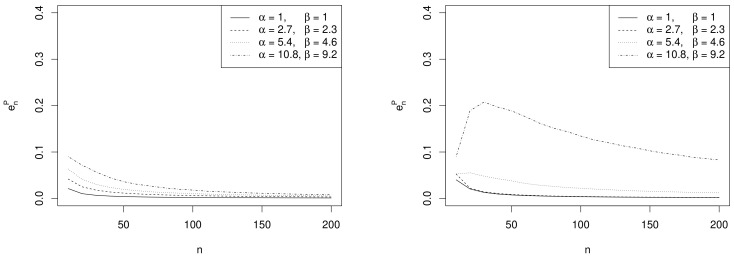
Plots of $e_{n}^{P}$ as a function of *n* for several values of the prior hyperparameters (α,β) with $\theta_{d}=0.45$ (**left panel**) and $\theta_{d}=0.8$ (**right panel**), when the logodds ψ is the parameter of interest.

One of the drawbacks of approximate intervals for θ is that it is not guaranteed that $\left(\tilde{\ell },\tilde{u}\right)\subseteq \left[0,1\right]$. A common solution in the applications is to trasform the parameter into the log odds scale so that the normal approximation of the posterior improves. As an example we implemented the credible intervals introduced in Section 3.2. Figure 3 shows the behavior of $e_{n}^{P}$ as a function of *n* for the same choices of hyperparameters and design values used in the previous example. Similar remarks apply.

## 5. Conclusions

The control of relevant aspects of interval estimates is the starting point for the definition of several SSD criteria both from the frequentist and from the Bayesian perspective. For instance, in the Bayesian side, traditional criteria rely on the pre-posterior control of length and position of credible intervals. In this article we focus on a different request: we look for a sample size sufficiently large so that the approximate likelihood interval provides an accurate approximation to the HPD interval determined from the exact posterior distribution of the parameter of interest. Since the likelihood normal approximation does not depend on the prior distribution, another way to interpret the criterion is that it provides the smallest sample size such that the role of the prior in the posterior distribution is made negligible by the information provided by the data. This kind of analysis can be read in two different ways. On the one side, one can know the number of units needed to use safely closed-form and handy formulas (those provided by the normal approximation) in the place of exact Bayesian intervals. On the other hand, a data analyst who uses approximate intervals instead of exact Bayesian intervals can know the price of this choice in terms of expected discrepancy.

From another perspective this kind of preposterior analysis allows one to know what the study dimension should be for a consensus between a Bayesian interval and a frequentist interval, i.e., a non-informative analysis.

In general, the criterion we propose does not control the main goal of a clinical trial, that can be, for instance, accuracy of estimation or efficacy/inefficacy of a given treatment. For this reason, our criterion should be put beside additional criteria specifically related to the main goal of the trial. For instance in our examples of Section 4 we consider the optimal sample sizes based on ALC. Then, taking the maximum between the two sample sizes obtained using the two criteria, one can control both interval length and accuracy of approximation.

Possible extensions of this work are listed below.

1. Other models. The methodology proposed in the paper can be easily extended to other models and setups relevant to clinical trials applications. A natural extension is to two-arms designs for the comparison of two proportions (difference or log odds ratio), in which the additional issue of units allocation arises [32]. For a predictive approach to allocation based on the control of posterior variances, see for instance [33]. See also [5] for related ideas in the Poisson model.
2. Probability vs. Expectation. In Section 2.2 we propose to summarize the predictive distribution of the discrepancy using the expected value w.r.t. $f_{n}\left(\cdot |\theta_{d}\right)$. An alternative is to take into account the whole probability distribution of *P* and to determine the smallest *n* such that $P[P\left(X_{n}\right)>\epsilon_{P}]$ is sufficiently small.
3. Design prior. For simplicity in this article we have performed preposterior calculations using the sampling distribution $f_{n}\left(\cdot |\theta_{d}\right)$. An alternative is to consider the so-called *two–priors approach* [23,24,30,34]) which avoids local optimality by replacing the design value with the design prior.
4. Decision-theoretic approach. The approach proposed in the paper is performance-based. Alternatively one could follow some previous works and rephrase the problem in a decision-theoretic framework and define a measure of discrepancy based on the posterior expected loss of *C* and $\tilde{C}$. We will elaborate on this in the future.

## Figures and Tables

**Table 1:** Optimal sample sizes for several choices of the prior hyperameters and of the design values, given $\epsilon_{P}=\epsilon_{B}=0.01$ and $\epsilon_{L}=0.1$.

$\theta_{d}$	(α,β)	(1,1)	(2.7,2.3)	(5.4,4.6)	(10.8,9.2)
0.45	$n_{P}^{☆}$	49	80	119	182
	$n_{B}^{☆}$	42	96	180	347
	$n_{L}^{☆}$	265	262	257	247
	$n_{\tilde{L}}^{☆}$	267	267	267	267
0.80	$n_{P}^{☆}$	35	118	646	2911
	$n_{B}^{☆}$	91	228	482	992
	$n_{L}^{☆}$	170	169	169	167
	$n_{\tilde{L}}^{☆}$	172	172	172	172

## Data Availability

No new data were created or analyzed in this study. Data sharing is not applicable to this article.

## References

[1] Lee J., Chu C.T. (2012). Bayesian clinical trials in action. Stat. Med..

[2] Yin G., Lam C.K., Shi H. (2017). Bayesian randomized clinical trials: From fixed to adaptive design. Contemp. Clin. Trials.

[3] Bittl J.A., He Y. (2017). Bayesian analysis. A practical approach to interpret clinical trials and create clinical practice guidelines. Circ. Cardiovasc. Qual. Outcomes.

[4] Spiegelhalter D.J., Abrams K.R., Myles J.P. (2004). Bayesian Approaches to Clinical Trials and Health-Care Evaluation.

[5] De Santis F., Gubbiotti S. (2019). A note on the progressive overlap of two alternative Bayesian intervals. Commun. Stat. Theory Methods.

[6] Lesaffre E., Lawson A.B. (2012). Bayesian Biostatistics.

[7] Gelman A., Carlin J.B., Stern H.S., Dunson D.B., Vehtari A., Rubin D.B. (2013). Bayesian Data Analysis.

[8] Kalbfleisch J.G. (1985). Probability and Statistical Inference. Volume 2: Statistical Inference.

[9] Robert C.P. (2007). The Bayesian Choice: From Decision-Theoretic Foundations to Computational Implementation.

[10] Meeker W.Q., Hahn G.J., Escobar L.A. (2017). Statistical Intervals: A Guide for Practitioners and Researchers.

[11] Brutti P., De Santis F., Gubbiotti S. (2014). Bayesian—Frequentist sample size determination: A game of two priors. Metron.

[12] Adcock C.J. (1997). Sample size determination: A review. J. R. Stat. Soc. Ser. D Stat..

[13] Joseph L., du Berger R., Belisle P. (1997). Bayesian and mixed Bayesian/likelihood criteria for sample size determination. Stat. Med..

[14] Joseph L., Wolfson D. (1997). Interval-based versus decision theoretic criteria for the choice of sample size. J. R. Stat. Soc. Ser. Stat..

[15] Brutti P., De Santis F. (2008). Robust Bayesian sample size determination for avoiding the range of equivalence in clinical trials. J. Stat. Plan. Inference.

[16] Cao J., Lee J.J., Alber S. (2009). Comparison of Bayesian sample size criteria: ACC, ALC, and WOC. J. Stat. Plan. Inference.

[17] Gubbiotti S., De Santis F. (2011). A Bayesian method for the choice of the sample size in equivalence trials. Aust. New Zealand J. Stat..

[18] Joseph L., Wolfson D.B., Berger R.D. (1995). Sample Size Calculations for Binomial Proportions via Highest Posterior Density Intervals. J. R. Stat. Soc. Ser. D Stat..

[19] M’Lan C.E., Joseph L., Wolfson D.B. (2008). Bayesian sample size determination for binomial proportions. Bayesian Anal..

[20] De Santis F., Fasciolo M.C., Gubbiotti S. (2013). Predictive control of posterior robustness for sample size choice in a Bernoulli model. Stat. Methods Appl..

[21] De Santis F. (2006). Sample size determination for robust Bayesian analysis. J. Am. Stat. Assoc..

[22] Brutti P., De Santis F., Gubbiotti S. (2008). Robust Bayesian sample size determination in clinical trials. Stat. Med..

[23] Joseph L., Belisle P. (2019). Bayesian consensus-based sample size criteria for binomial proportions. Stat. Med..

[24] Brutti P., De Santis F., Gubbiotti S. (2014). Predictive measures of the conflict between frequentist and Bayesian estimators. J. Stat. Plan. Inference.

[25] De Santis F., Gubbiotti S. (2017). A decision-theoretic approach to sample size determination under several priors. Appl. Stoch. Model. Bus. Ind..

[26] Casella G., Berger R. (2001). Statistical Inference.

[27] R Core Team (2018). R: A Language and Environment for Statistical Computing.

[28] Sacchi S., Marcheselli R., Bari A., Buda G., Molinari A.L., Baldini L., Vallisa D., Cesaretti M., Musto P., Ronconi S. (2016). Safety and efficacy of lenalidomide in combination with rituximab in recurrent indolent non-follicular lymphoma: Final results of a phase II study conducted by the Fondazione Italiana Linfomi. Haematologica.

[29] Zhou H., Lee J.J., Yuan Y. (2017). BOP2: Bayesian optimal design for phase II clinical trials with simple and complex endpoints. Stat. Med..

[30] Sambucini V. (2019). Bayesian predictive monitoring with bivariate binary outcomes in phase II clinical trials. Comput. Stat. Data Anal..

[31] Morita S., Thall P.F., Muller P. (2008). Determining the effective sample size of a parametric prior. Biometrics.

[32] M’Lan C.E., Joseph L., Wolfson D.B. (2006). Bayesian Sample Size Determination for Case-Control Studies. J. Am. Stat. Assoc..

[33] De Santis F., Perone Pacifico M., Sambucini V. (2004). Optimal predictive sample size for case-control studies. Appl. Stat..

[34] Wang F., Gelfand A.E. (2002). A simulation-based approach to Bayesian sample size determination for performance under a given model and for separating models. Statist. Sci..

